# Colovesical fistula causing an uncommon reason for failure of computed tomography colonography: a case report

**DOI:** 10.1186/1752-1947-6-214

**Published:** 2012-07-23

**Authors:** Angeliki Neroladaki, Romain Breguet, Diomidis Botsikas, Sylvain Terraz, Christoph D Becker, Xavier Montet

**Affiliations:** 1 Department of Radiology, Geneva University Hospital, Gabrielle-Perret-Gentil, 4, 1211, Geneva 4, Switzerland

## Abstract

**Introduction:**

Computed tomography colonography, or virtual colonoscopy, is a good alternative to optical colonoscopy. However, suboptimal patient preparation or colon distension may reduce the diagnostic accuracy of this imaging technique.

**Case presentation:**

We report the case of an 83-year-old Caucasian woman who presented with a five-month history of pneumaturia and fecaluria and an acute episode of macrohematuria, leading to a high clinical suspicion of a colovesical fistula. The fistula was confirmed by standard contrast-enhanced computed tomography. Optical colonoscopy was performed to exclude the presence of an underlying colonic neoplasm. Since optical colonoscopy was incomplete, computed tomography colonography was performed, but also failed due to inadequate colon distension. The insufflated air directly accumulated within the bladder via the large fistula.

**Conclusions:**

Clinicians should consider colovesical fistula as a potential reason for computed tomography colonography failure.

## Introduction

Computed tomography colonography (CTC) or virtual colonoscopy has gained wide acceptance and is now considered as an excellent alternative in case of incomplete or contraindicated conventional optical colonoscopy (OC) [1]. However, suboptimal patient preparation or suboptimal colon distension may reduce the diagnostic accuracy of CTC.

Even though CTC is a well-recognized method for colon analysis and detection of lumen abnormalities, some pitfalls and limitations may prevent an adequate bowel analysis. A few other publications have reported colovesical fistula diagnosed with CTC, but with adequate distension of the colon [2,3]. To the best of our knowledge, we report the second case of inadequate colon distension due to an important colovesical fistula in the context of colonic diverticulitis [4].

## Case presentation

**Figure 1:**
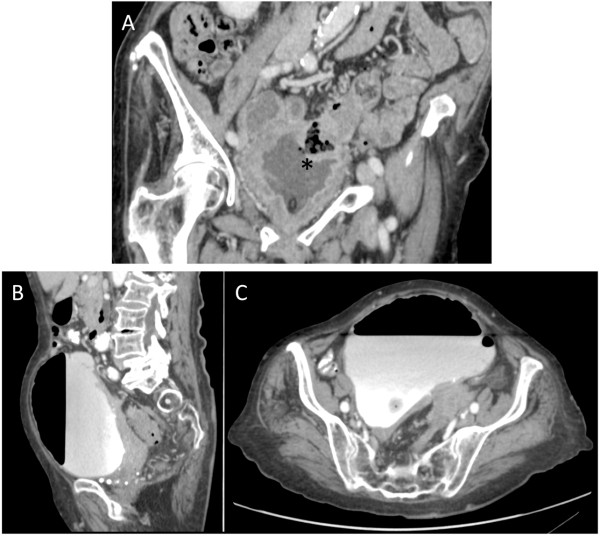
**(A) Coronal reformatted CT acquired before the CTC shows an obvious colovesical fistula (asterisk).(B,C)** After insertion of a Foley catheter and insufflation of air in the rectum, a great quantity of air appeared in the bladder, without colon distension

An 83-year-old Caucasian woman presented with a five-month history of pneumaturia and fecaluria and an acute episode of macrohematuria, leading to a high clinical suspicion of a colovesical fistula. Our patient also reported a weight loss of 5kg in a short period of time but had no other significant clinical history. The initial contrast-enhanced computed tomography (CT) clearly depicted the suspected colovesical fistula (Figure 1).

An OC was performed to investigate the colon for the potential presence of a colic neoplasm associated with the colovesical fistula. The OC was incomplete and progressed to only 35cm from the anal margin due to bad tolerance and a local inflammatory response related to severe diverticulosis.

CTC was requested after this incomplete OC. Our patient received a low residue diet for 48 hours and a 24-hour clear liquid diet prior to the CTC. Laxatives were also administered to allow colonic evacuation, as well as a stool marker for fecal tagging 24 hours prior to the CTC. Our patient was positioned in the left decubitus position and air was manually insufflated through an intrarectal small gauge Foley catheter according to our patient’s tolerance. Since the first scout image (with our patient in a supine position) revealed inadequate bowel distension, insufflations were continued and a second scout showed the presence of air in the pelvis but no colonic distension. Biphasic CT was performed, with our patient first in a prone position, then in a supine position. The second acquisition was realized after injection of 120mL of iodinated contrast media (Accupaque 350, GE Healthcare, Glattbrugg, Switzerland). The CT parameters were as follows: 120kV, 120mA (modulated) and 2mm slice thickness with an interval of 1mm, corresponding to a dose length product of 206mGy·cm for the non-enhanced phase and 436mGy·cm for the contrast-enhanced phase.

The images acquired showed a significant distension of her bladder, with an estimated volume of 1,350mL and filled with contrast, and the presence of an intravesical air-fluid level. There was no colonic distension. The two-dimensional multiplanar analysis of the images successfully revealed the fistulous tract between her bladder and her sigmoid colon but an analysis of her bowel lumen was not performed due to inadequate colonic distension (see Figure 1). Cystoscopy revealed a vesical opening with fecal material leakage 2cm to 3cm superior to the left ureterovesical junction, with no extrinsic visible lesion. Our patient underwent a sigmoidectomy with resection of the fistulous tract. Histopathology revealed a severe diverticulosis with focal diverticulitis of her sigmoid colon that had perforated into her bladder. Fibrotic tissue was present, but no neoplastic cells were identified.

## Discussion

CTC is a robust imaging technique that is able to visualize the lumen of the colon and to detect the presence of polyps and masses. Thus, CTC could be a suitable alternative if OC is impossible or incomplete.

OC is not accepted by all patients and has several contraindications, such as severe comorbidity, bleeding disorders, advanced age, allergic reaction to anesthesia or sedation [1]. As many as 6% to 26% of examinations performed are incomplete and fail to reach the caecum [5]. The reasons for incomplete OC may be colonic redundancy, angulation and fixation of colonic loops, severe diverticulitis, adhesions and spasms as well as insufficient preparation. In these cases or if OC is not technically feasible, CTC in now increasingly performed.

In order to be successful, CTC requires adequate patient preparation and data acquisition, as well as imaging assessment including both two-dimensional multiplanar and virtual three-dimensional endoscopic images. Under optimal conditions, that is, a clean, dry and completely distended colon, without CT artefacts, CTC has similar detection rates for advanced neoplasia to OC [6]. Some authors have demonstrated that CTC is also feasible in a non-cathartic-prepared colon with a performance similar to OC, at least in a population with a high prevalence of neoplasms (for neoplasms of at least 6mm diameter) [7]. Adding CTC after incomplete OC allows clinicians to obtain complete examination of the colon in 91% of cases [5].

The majority of technical errors arise from residual stool and fluid, colic spasms and insufficient insufflations, as well as respiratory, stair steps and metallic artefacts [8]. When cleansing or distension of the colon is suboptimal, the rate of false positives or false negatives increases, leading to a decreased diagnostic performance of CTC.

Sufficient bowel distension is crucial for the accurate assessment of intraluminal colonic defects such as polyps. An underinflated bowel has luminal narrowing and some colonic segments are collapsed, leading to interpretation errors. Finally, CTC images are acquired in both supine and prone positions, in order to shift the position of fluid and retained feces and to visualize more accurately the nondependent mucosal-luminal interface of all colonic segments.

## Conclusion

We report here a case of unsuccessful CTC due to insufficient colon distension related to a colovesical fistula. Colonic distension failed because all the insufflated air accumulated in the bladder. Clinicians have to be aware that a colovesical fistula may preclude successful CTC and thus may be a relative contraindication to CTC.

## Consent

Written informed consent was obtained from the patient for publication of this case report and any accompanying images. A copy of the written consent is available for review by the Editor-in-Chief of this journal.

## Competing interests

The authors declare that they have no competing interests.

## Authors’ contributions

AN, RB and XM were directly involved in the management of the patient. AN, RB and XM wrote the manuscript with support from DB and ST. CDB and XM reviewed and interpreted the CT colonography images. All authors read and approved the final manuscript.
